# Paclitaxel and mortality in patients with claudication and de novo femoropopliteal lesions: a historical cohort study

**DOI:** 10.1186/s42155-021-00255-1

**Published:** 2021-08-23

**Authors:** Gérald Gahide, Samuel C. Phaneuf, Mathilde Cossette, Amine Banine, Martina Budimir, Kourosh Maghsoudloo, Phillip Fei, Bo Yi Dou, Maxime Bouthillier, Charles Alain, Simon Bradette, Maxime Noel-Lamy, Francois Belzile, Bao The Bui, Marc Antoine Despatis, Jean Francois Vendrell

**Affiliations:** 1 Service de Radiologie Interventionnelle. Département d’Imagerie Médicale, Centre Hospitalier Universitaire de Sherbrooke, 3001, 12ème Avenue Nord, Sherbrooke, Québec J1H 5H3 Canada; 2 Centre de Recherche du CHUS, Etienne Le Bel, Université de Sherbrooke, 12e Avenue Nord Porte 6, Sherbrooke, Québec J1H 5N4 Canada; 3 Faculté de Médecine et des Sciences de la Santé, Université de Sherbrooke, 3001 12 ème Avenue Nord Immeuble X1, Sherbrooke, Québec J1H 5N4 Canada; 4 Service de Chirurgie Vasculaire, Département de Chirurgie, Centre Hospitalier Universitaire de Sherbrooke, 3001, 12ème Avenue Nord, Sherbrooke, Québec J1H 5H3 Canada

**Keywords:** Peripheral artery disease, Claudication, Paclitaxel, Mortality, Drug eluting stent, Drug coated balloon

## Abstract

**Objective:**

To compare the mortality rates of patients with claudication and de novo femoropopliteal lesions treated with and without paclitaxel coated devices (PCD).

**Background:**

A recent meta-analysis, mostly including patients with claudication and de novo femoropopliteal lesions but also with recurrent stenoses and critical limb ischemia, has shown a significant excess mortality in patients treated with PCD.

**Methods:**

Comparison of two historical cohorts of patients presenting with claudication and de novo femoropopliteal lesions treated with and without PCD between 2008 and 2018.

**Results:**

After review of 5219 arteriograms in patients presenting with peripheral artery disease, 700 consecutive patients were included consisting in 72.6% of male (*n* = 508). Mean age was 68.1 ± 8.5 years. 45.7% of the patients (*n* = 320) had a treatment including a PCD. Mean femoropopliteal lesion length was 123 ± 91 mm including 44.6% of occlusions. Patients of the control group were censored at crossover to paclitaxel when applicable. Mortality rates at 1, 2 and 5 years were 4.6%, 7.5%, 19.4% and 1.6%, 6.2%, 16.6% in the non-PCD and PCD groups respectively. The relative risks of death when using PCD were 0.35 (*p* = 0.03), 0.83 (*p* = NS) and 0.86 (p = NS) at 1, 2 and 5 years respectively.

**Conclusion:**

There was no excess mortality in patients with claudication and de novo femoropopliteal lesions treated with paclitaxel coated devices at 1, 2 and 5 years of follow-up in this cohort. The current study suggests that additional prospective randomized studies properly powered to study mortality are necessary.

## Background

The development of paclitaxel-coated devices (PCD) has allowed physicians to increase the proportion of patients treated with primary balloon angioplasty without the need of secondary stenting as this new approach was associated with a dramatic improvement in long term patency (Laird et al. 2019). A meta-analysis published by Katsanos et al. in 2018 has had a profound impact upon the vascular community (Katsanos et al. 2018). In that study, the authors reported a significant absolute increase of 3.4% of all cause death at 2 years and 6.6% at 5 years in comparison to the control group when using PCD. Following this publication, the multidisciplinary VIVA physician group did an impressive work in close collaboration with industry. They comprehensively re-analyzed the data of 8 prospective randomized clinical trials (Rocha-Singh et al. 2020). After this thorough reanalysis, they demonstrated a signal of excess mortality at 5 years in the PCD group. It is of importance to note that both meta-analyses were based upon studies that included patients with de novo claudication, recurrent stenoses and critical limb ischemia.

Following this so-called Paclitaxel controversy, the FDA recommended to consider alternative treatment options to PCD and to continue diligent monitoring of patients who have been treated with paclitaxel-coated balloons and paclitaxel-eluting stents (UETMIS n.d.). Given the large use of this very promising technology, it was chosen to review the entire database to compare two historical treatments. This study aimed at comparing the 1 year, 2 years and 5 years mortality rates of patients with claudication and de novo femoropopliteal lesions treated endovascularly with PCD in comparison to a historical cohort treated without PCD.

## Material and methods

### Study population

The study was approved by the local institutional ethics committee of the *Centre Intégré Universitaire de Santé et de Services Sociaux de l’Estrie - Centre Hospitalier Universitaire de Sherbrooke, Quebec, Canada* (CIUSSS de l’Estrie-CHUS). This study was a single-center observational retrospective review of consecutive patients treated with plain old balloon ± bare metal stent (POBA) or Paclitaxel Coated Device (PCD) including Drug Coated Balloon (DCB) and Drug Eluting Stents (DES) for the treatment of disabling intermittent claudication at the University Hospital of Sherbrooke from January 2008 to December 2018. The written consent to participate in the study was waived due to its retrospective character. Following the institutional and local regulatory policies, all patients signed written informed consent before undergoing the procedure.

A review of the entire database of patients treated endovascularly during the 2008–2018 period at the institution was performed. Consecutive patients who presented with lifestyle limiting claudication and have had an endovascular treatment of de novo femoropopliteal disease were included.

Inclusion criteria were as follows: 1- patients older than 18y-o; 2- presenting with disabling intermittent claudication (*Rutherford stage 2 and 3*); 3- referred for endovascular revascularization of de novo femoropopliteal artery lesions. Exclusion criteria were as follows: 1-Past history of femoropopliteal bypass graft; 2- Past history of endovascular revascularization of ipsilateral or contralateral femoropopliteal artery lesion(s); 3- Past or current history of critical limb ischemia (Rutherford class 4, 5 and 6).

Detailed demographic, clinical, and procedural informations were gathered for each patient from their electronic medical record. Patients treated with a DCB and/or DES were enrolled in the PCD group. Patients treated with a POBA and/or BMS were enrolled in the POBA group. It was imperative for patients in the POBA group not to have been in contact with a PCD neither before the index procedure nor during the follow-up. When a patient initially in the POBA group was subsequently treated with PCD, the follow-up was censored at the date of this crossover to paclitaxel treatment.

### Study endpoints

The primary endpoint of this study was the difference in all-cause mortality rates after POBA and PCD angioplasty at 1, 2 and 5-years of follow-up. Secondary endpoints included the relationship between paclitaxel dose and mortality, and search for mortality predictors, including type of treatment (POBA vs. PCD), age, sex, dyslipidemia, diabetes mellitus, tobacco use, renal insufficiency, cancer, chronic obstructive pulmonary disease, coronary artery disease and heart failure.

### Data collection

**Fig. 1:**
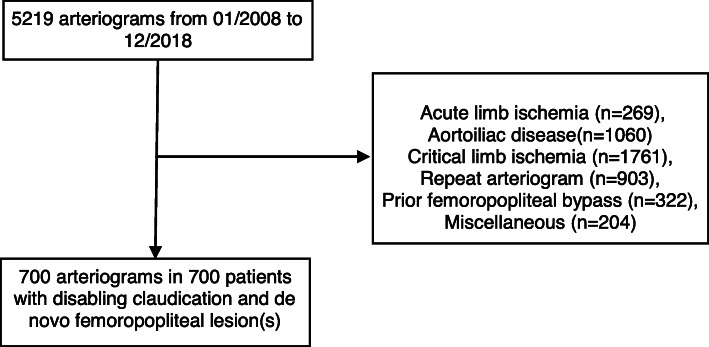
Flow chart of the selection of the patients included in the study

Since 1995, all clinical and biological files of the patients treated are systematically and comprehensively gathered in an Electronic Health Record system. Since 2000, all the imaging files are archived in a PACS system. Paclitaxel Coated Devices are used as a standard of practice for the treatment of femoropopliteal artery lesions since 2014 (UETMIS n.d.). A systematic review of the entire database of lower limb arteriograms performed from January 2008 to December 2018 was conducted. After careful review and analysis of the clinical indications and treatments, 700 patients were included (cf. flow chart Fig. 1). The clinical characteristics and risk factors of the patients were extracted from their numerical files. The reported baseline clinical characteristics were those present at the time of treatment. The corresponding 700 DSA were carefully analyzed to determine arterial lesion lengths, locations and quality (stenosis or occlusion). When applicable, the date of death of the patients were retrieved from a registry held by the Régie de l’Assurance Maladie du Quebec, the organism responsible for the management of public health in the province of Québec. All the death occurring in the province are gathered in this database. The interrogation date of this mortality registry for the current study was the 14th of October 2020.

### Statistical analysis

Continuous variables were expressed as means ± standard deviation (SD). Dichotomous and categorical variables were expressed as counts and percentages. Continuous variables were compared using the Mann-Whitney or Student’s t-tests. Categorical data were compared using the chi-squared or Fisher’s exact tests. Survival was evaluated using Kaplan-Meier analysis; the survival curves were compared using the log-rank test. Between-group comparisons were.

made with a time-dependent multivariable Cox regression model in order to adjust group hazard mortality ratios with known confounding factors. This time-varying model allowed to consider the patients included in the POBA group that were subsequently treated with PCD without censoring them. The Cox model was adjusted for age, sex, active tobacco use, diabetes, cancer, hyperlipidemia, arterial hypertension, Charlson score and heart failure. The level of statistical significance was set at *p* < 0.05. The software used was IBM SPSS Statistics, v.24, Armonk, NY.

## Results

### Patients characteristics

From 2008 to 2018, a total of 700 consecutive patients presenting with lifestyle limiting claudication and treated for de novo femoropopliteal artery disease were included. The cohort included 72.6% of male (*n* = 508). Mean age was 68.1 ± 8.5 years.

All baseline demographics and clinical characteristics were summarized on a patient-basis (cf. Table 1). Cardiovascular risk factors were highly prevalent, including arterial hypertension in 51.3%, hyperlipidemia in 50%, smoking in 19.4%, and diabetes in 27.4% of patients. Several baseline characteristic differences were statistically significant between the POBA and DCB groups, respectively: Diabetes mellitus [31.1% and 23.1% (*p* = 0.02)], Dyslipidemia [59.2% and 39.1% (*p* < 0.001)]; Coronary Artery Disease [44.2% and 34.3% (p = < 0.001)], Tobacco use [24.3% and 14.7% (*p* = 0.004)]; Arterial Hypertension [58.9% and 42.2% (p < 0.001)], COPD [15% and 9.4% (*p* = 0.03)], and Charlson score [0.44 ± 0.98 and 0.31 ± 0.9 (*p* = 0.04)].

**Table 1:** Comparison of the baseline characteristics of the patients in the PCD and POBA groups and the 1, 2 and 5 years mortality rates

	POBA (*n* = 380)	PCD (*n* = 320)	*p*-value
Age	67.7y + −8.8	68.5y + − 8.2	*p* = 0.14
Female	27.1% (*n* = 103)	27.8% (*n* = 89)	*p* = 0.835
Active Tobacco Use	23.4% *n* = (89)	14.7% (*n* = 47)	*p* = **0.004**
Cancer	10.5% *n* = (40)	10.3% (*n* = 33)	*p* = 0.93
Creatinin	92.8 + −81.0	88.7 + −68.7	*p* = 0.57
Renal Insufficiency	8.4% (n = 32)	7.5% (*n* = 24)	*p* = 0.65
Diabetes melllitus	31.1% *n* = (118)	23.1% (n = (74)	*p* = **0.019**
Hyperlipidemia	59.2% *n* = (225)	39.1% (*n* = 125)	*p* < **0.001**
Hypertension	58.9% *n* = (224)	42.2% (*n* = 135)	*p* < **0.001**
Heart failure	2.9% *n* = (11)	1.3% (*n* = 4)	*p* = 0.13
Coronary Artery Disease	44.2% *n* = (168)	34.3% (*n* = 97)	*p* < **0.001**
COPD	15% *n* = (57)	9.4% (*n* = 30)	*p* = **0.03**
Charlson Score	0.44 ± 0.98	0.31 ± 0.9	*p* = **0.04**
1 year mortality rate	4.6%	1.6%	*p* = **0.03**
2 year mortality rate	7.5%	6.2%	*p* = 0.53
5 year mortality rate	19.4%	16.6%	*p* = 0.49

### Treatments

The mean dose of paclitaxel delivered per intervention in the PCD group was 12.3 ± 9.4 mg. Details regarding the paclitaxel coated devices used are reported in Table 2. 140 patients had same day bilateral femoropopliteal treatments. There were 44.8% of femoropopliteal occlusions (*n* = 376/840). Mean length of treated lesions were 123 ± 91 mm. Details regarding the femoropopliteal lesions are reported in Table 3.

**Table 2:**
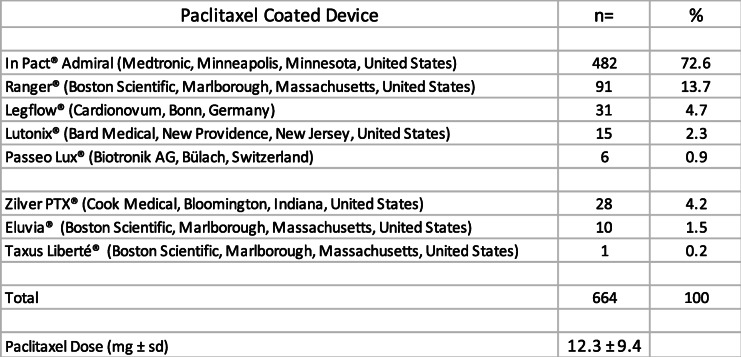
Type of the paclitaxel coated devices used in the PCD group and paclitaxel mean dose

**Table 3:** Characteristics of the femoropopliteal lesions in both PCD and POBA groups

	POBA (*n* = 380)	PCD (*n* = 320)	*p*-value
Mean Lesion Length (mm)	111 + −89	135 + −91	*p* < **0.001**
Occlusion	40.2% (*n* = 177)	50% (*n* = 199)	*p* = **0.005**
De Novo Lesion	100% (n=)	100% (n=)	N/A
Reccurent lesion	0% (*n* = 0)	0% (*n* = 0)	N/A
Prior Femoropopliteal Bypass	0% (*n* = 0)	0% (*n* = 0)	N/A
Bilateral Treatment	16.3% (*n* = 62)	24.3% (*n* = 78)	*p* < **0.01**

### Mortality study

**Fig. 2:**
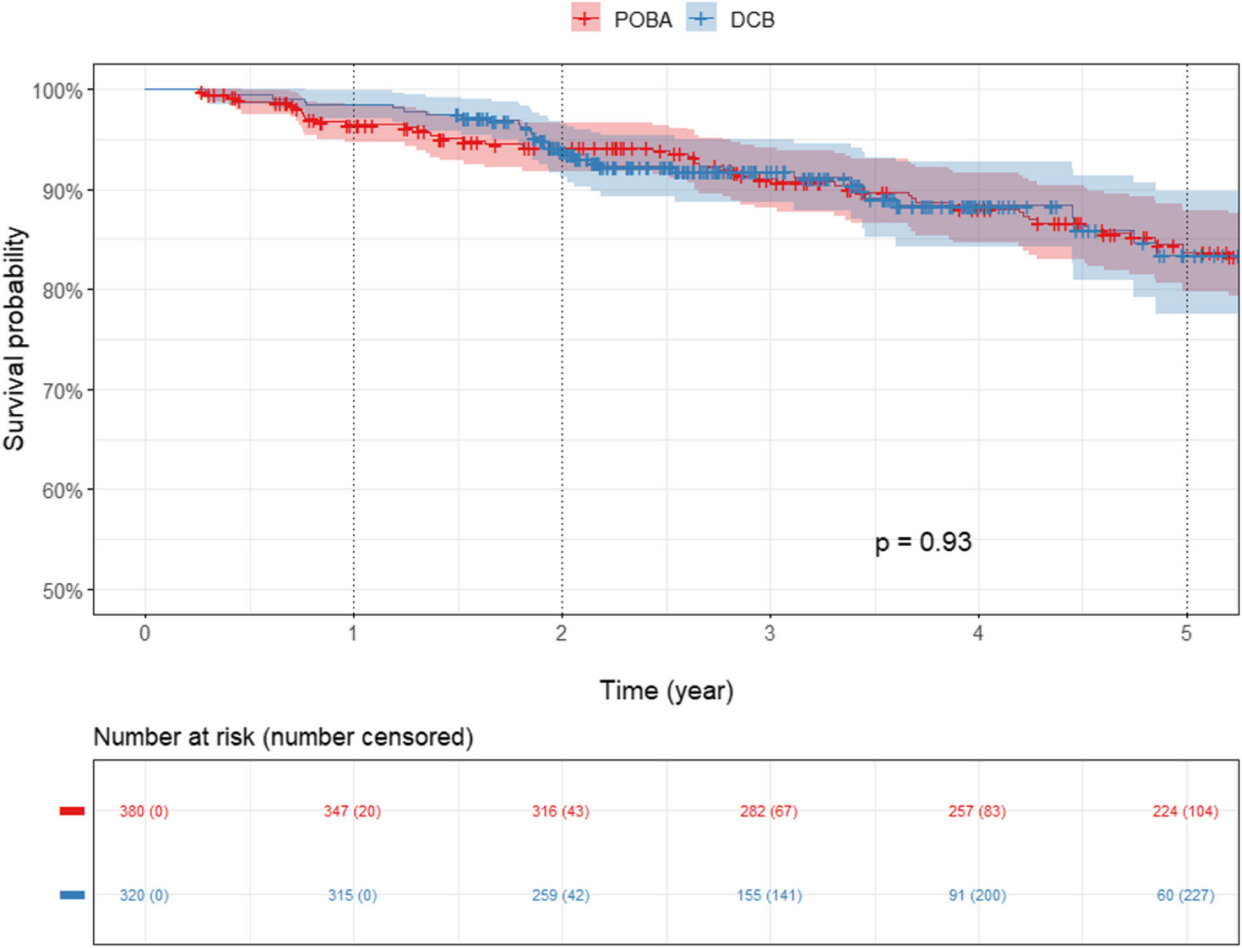
Kaplan-Meier estimates of survival of the entire cohort for the POBA and DCB group through 5 years including the interval of confidence. (PCD: Paclitaxel Coated Devices; POBA: Plain Old Balloon Angioplasty)

**Fig. 3:**
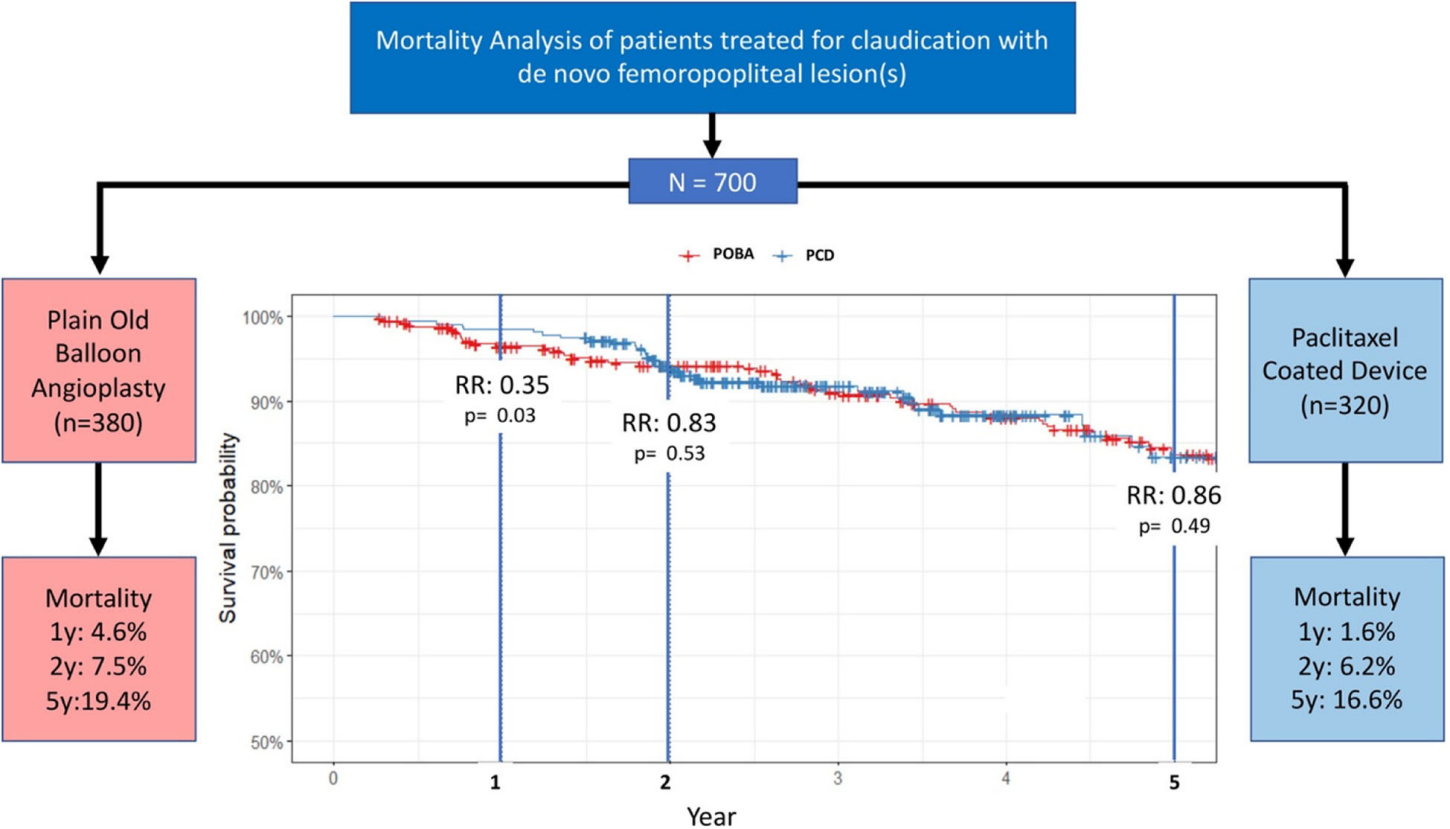
Annotated Kaplan-Meier survival analysis with the relative risk-ratio (RR) at 1, 2 and 5 years of follow-up. (PCD: Paclitaxel Coated Devices; POBA: Plain Old Balloon Angioplasty)

Mortality rates at 1, 2 and 5 years in the POBA and PCD groups were 4.6% CI [2.1–7.1], 7.5% [4.4–10.5], 19.4% [14.5–24] and 1.6% [0.2–2.9], 6.2% [3.5–8.9], 16.6% [10.2–22.6] respectively (*p* = 0.03 at 1 year and NS at 2 and 5 years). In comparison to the POBA group, the relative risks of death in the PCD group were 0.35, 0.83 and 0.86 at 1, 2 and 5 years. Kaplan Meier survival curves are presented in Fig. 2 and Fig. 3.

The multivariable Cox regression analysis showed that age (*p* < 0.0001), Tobacco use (*p* = 0.0003) and a Charlson score ≥ 1 (*p* < 0.02) were independent predictors of all-cause mortality. There were also trends for cancer (*p* = 0.053) and male sex (*p* = 0.052) (cf. Table 4). When adjusting for potential confounders and accounting for cross-over, PCD didn’t show any significant association with all-cause mortality. There was no correlation between DCB length or paclitaxel dose with mortality. Neither were there any correlation between treated lesion length and mortality.

**Table 4:** Multivariable Cox Regression Analysis for defining the independent factors of all cause death of the entire cohort of patients presenting with claudication and de novo femoropopliteal lesion(s)

Variables		Multivariate Analysis			
		Hazard Ratio	95% Confidence Interval		***p***-value
			Lower Limit	Upper Limit	
**Paclitaxel Coated Device**	Present vs Absent	0.9693	0.6943	1.353	0.855
**Age**		1.0720	1.0489	1.096	**<.0001**
**Sex**	Women vs Men	0.6719	0.4499	1.003	0.052
**Active Tobbaco Use**	Present vs Absent	2.0642	1.3917	3.062	**0.0003**
**Diabetes**	Present vs Absent	1.2256	0.8496	1.768	0.28
**Cancer**	Present vs Absent	1.5511	0.9934	2.422	0.053
**Hyperlipidemia**	Present vs Absent	1.1638	0.7812	1.734	0.46
**Arterial Hypertension**	Present vs Absent	0.8990	0.6144	1.315	0.58
**Heart Failure**	Present vs Absent	1.1566	0.4141	3.230	0.78
**Charlson score**	1 vs 0	2.5876	1.5844	4.226	**0.0001**
	2 vs 0	3.6846	1.2438	10.915	**0.019**

## Discussion

The current study, comparing two historical cohorts of patients with claudication and de novo femoropopliteal artery lesions treated with and without paclitaxel coated devices (PCD), failed to show any excess mortality with the use of PCD. On the contrary, there was a statistically significant excess mortality at 1 year of follow-up in the non PCD group. This excess mortality was not maintained over time at 2 and 5 years of follow-up. Due to the historical cohort design of our study, with most patients treated with POBA prior to 2014 and with DCB afterwards, it cannot be ruled out that a potential deleterious effect of PCD may have been masked by improved medical management of the comorbidities. Nevertheless, those results are consistent with two recent studies that also failed to show any significant increase in mortality when using paclitaxel coated devices (Böhme et al. 2020; Nordanstig et al. 2020).

Despite its retrospective design, this work has several strengths. The availability of a national mortality registry limited the potential lost to follow-up bias. This allowed to draw reliable conclusions regarding the mortality of the patients included in this work. Furthermore, it was chosen to only include the patients who presented for their very first endovascular treatment of femoropopliteal artery related disabling claudication. This is important because patients with recurring disease are by definition older and have a more aggressive atherosclerotic disease with a potentially higher mortality rate. Moreover, given the longitudinal design of this study, many patients with recurring disease were already included in the de novo claudication group. It is important to note that the patients in the POBA group had significantly higher cardiovascular risk factors such as tobacco use, hypertension, dyslipidemia and diabetes. Nevertheless, after adjustment for potential confounders and accounting for cross-over, PCD didn’t show any significant association with all-cause mortality.

On the other hand, patients in the PCD group had significantly longer lesions and more occlusions. These differences are very likely explained by the fact that the institutional technology evaluation unit recommended the use of PCD for TASC C and D femoropopliteal lesions but the choice between POBA and PCD was left at the operator’s discretion for TASC A and B lesions (UETMIS n.d.).

The question of how a supposedly local treatment may be responsible for an excess mortality 2 years and 5 years later is currently a conundrum. There are three potential explanations: a currently unknown physiopathological mechanism, randomness or a methodological mistake.

Regarding the potential physiopathological mechanism, several factors influence the fraction of drug released into the arterial wall and the fraction released in the systemic circulation, such as the drug carrier and the arterial lesion complexity (Tzafriri et al. 2010; Granada et al. 2019). Though there is an initial burst of serum paclitaxel following DCB or some of the DES delivery, this is a transient phenomenon with the drug being cleared during the following days (Scheinert et al. 2014; Dake et al. 2011). The rest of paclitaxel is receptor bound, and there is no evidence for a paclitaxel reservoir in the organism (Levin et al. 2004). A dose-effect relationship had been initially suspected but never confirmed in accordance with the current study. Performance and detection biases had been suspected since interventional studies are difficult to blind (Beckman and White 2019). One of the hypothesis was that the patients in the control arm, with more restenosis, might have had more visits with study investigators at which secondary prevention medical therapies could have been properly adjusted. Another hypothesis could be that the patients treated with PCD having a better ambulatory capability could have been more active, raising the risk of stress-induced myocardial infarction. Nevertheless, as of today, no study has demonstrated a significant increase in cardiovascular events in the paclitaxel group. It is also important to realize that the mechanism of action, if mechanism there is, could impact the way we should analyze the mortality. If the phenomenon precipitates some already preexisting conditions that were about to decompensate in the near future, then this premature death will be noticed only during a certain period of time. This mortality displacement or “harvesting effect” implies that after some periods with excess mortality, there is a decrease in overall mortality during a subsequent period of time (Saha et al. 2014). On the other hand if this is a toxic phenomenon, occurring randomly in patients or without regard to the underlying conditions, then the excess mortality should be maintained over a longer period of time. The current study refutes both hypotheses with on the contrary an excess mortality in the POBA group at 1 year of follow-up and almost no difference at 2 and 5 years of follow-up.

Patients with claudication are expected to benefit the most from drug coated devices. The longer patency rates associated with PCD are synonym with a better ambulatory capability and better quality of life. Both Katsanos et al. and Rocha-Singh et al. studies included mostly patients with claudication but also 11% and 6% of patients with CLI respectively. It is important to note that patients with critical ischemia have a higher mortality rate than patients with claudication, having been reported to be up to 36% at 2 years in CLI (Mustapha et al. 2018) while ranging between 4 and 10% in patients with claudication (Katsanos et al. 2018; Böhme et al. 2020). When combined together, the mortality rate of patients with de novo claudication and critical ischemia does not follow a normal distribution, but a bimodal distribution (i.e. a mortality curve with two peaks). The probability of death has a binomial distribution (dead or alive). The closer the probability of a binomial event is to 50%, the larger is its standard deviation and its confidence interval, meaning that the potential range of rate of death is larger than in a group with a smaller probability. The worst case scenario is when there is a mix with only a marginal number of patients with a higher mortality rate. Then the second peak may be hidden in the right part of the main curve, but nevertheless dramatically impacting the overall mortality in one way or the other. The meta-analysis by Katsanos et al., included 89% (*n* = 4133) of patients with claudication and only 11% (*n* = 530) with CLI. At 2 years the 95% confidence interval of potential number of deaths ranges between 141 and 190 among the patients with claudication while it may range between 169 and 213 for the patients with CLI (i.e. more than half of the expected deaths). This shows how an apparently small proportion of 11% of patients with CLI may have a huge impact on mortality rates in one way or the other, not allowing to draw any reliable statistical conclusion.

Consequently, both groups of patients with claudication and CLI should be studied separately as in the study by Nordanstig et al. The same principle applies to patients with recurring claudication who should also be studied separately. It could be relevant for both the VIVA group and Katsanos et al. to report the respective mortality rates of patients with de novo claudication, recurrent disease and critical limb ischemia to better appreciate the impact of each group on mortality analyses.

Given the large use of this very promising technology that significantly improved patency rates in comparison to POBA, the study by Katsanos et al. has had an important impact upon endovascular practice with PCD taken off the shelves in many centers meanwhile reaching a definitive conclusion regarding its potential deleterious effect. Although claudication may be a severely debilitating disease with dire consequences for the quality of life of patients, the possibility of increasing the risk of death dramatically lowered its benefit to risk ratio. On the other hand, given the lack of physiopathological mechanism, and knowing that the meta-analysis combined studies that were not powered to study mortality, it was very difficult to offer a second class treatment with all its limitations instead of drug eluting devices that would provide improved patency rates, lower reinterventions and better quality of life. In order to help us with this ethically demanding task, the FDA recently asked the Multi-Specialty and Multi-Society Coalition for Patient Safety with Paclitaxel Technologies to develop a set of talking points concerning the risks and benefits of using paclitaxel devices (Multi-Specialty and Multi-Society Coalition for Patient Safety With Paclitaxel Technologies Talking Points Document n.d.). Among others, the committee indicated that in individual patients judged to be at particularly high risk for restenosis and repeat femoropopliteal interventions, clinicians may determine that the benefits of using a paclitaxel-coated device may outweigh the risk of late mortality. The results of the current study, that failed to show any increase in mortality in patients with de novo claudication treated with paclitaxel coated devices, support the need for additional prospective randomized studies properly powered to study mortality.

## Conclusion

The paclitaxel controversy has cast doubts over the safety profile of paclitaxel for the management of peripheral artery disease in patients with claudication and de novo femoropopliteal lesions. The current study, including a large group of patients with claudication and de novo femoropopliteal lesions didn’t show any excess mortality with the use of paclitaxel up to 5 years of follow-up and support the need for additional prospective randomized studies properly powered to study mortality.

## Limitations

This was a retrospective study comparing two historical cohorts with the potential biases related to this methodology.

Though one strength of this study was the availability of a provincially held register of patients’ death, limiting the potential lost to follow-up patients, the specifics regarding the causes of death weren’t available.

The POBA arm was recruited from 2008 to 2018 and the PCD was recruited from 2014 to 2018.

### Key results

- Mortality rates at 1, 2 and 5 years were 4.6%, 7.5%, 19.4% and 1.6%, 6.2%, 16.6% in the Plain Old Balloon and the Paclitaxel Coated Device groups respectively.

- The relative risks of death when using paclitaxel coated devices were 0.35 (*p* = 0.03), 0.83 and 0.86 (p = NS) at 1, 2 and 5 years respectively.

### Implications for patient care

**-** There was no excess mortality at 1, 2 and 5 years of follow-up with the use of paclitaxel coated devices in patients with claudication and de novo femoropopliteal lesions.

**-**The current study suggests that additional prospective randomized studies properly powered to study mortality are necessary.

### Summary statement

There was no excess mortality in this cohort at 1, 2 and 5 years of follow-up when using paclitaxel coated devices in patients with claudication and de novo femoropopliteal lesions.

### Type of research

Single-center retrospective observational study.

## Data Availability

Not applicable.

## Abbreviations

CLI: Critical Limb Ischemia
DCB: Drug Coated Balloon
DES: Drug Eluting Stent
PCD: Paclitaxel Coated Devices
POBA: Plain Old Balloon Angioplasty
